# A novel framework based on explainable AI and genetic algorithms for designing neurological medicines

**DOI:** 10.1038/s41598-024-63561-3

**Published:** 2024-06-04

**Authors:** Vishakha Singh, Sanjay Kumar Singh, Ritesh Sharma

**Affiliations:** 1 Department of Computer Science and Engineering, Indian Institute of Technology (BHU), Varanasi, 221005 Uttar Pradesh India; 2 Department of ICT, Manipal Institute of Technology, Manipal, 576104 Uttar Pradesh India

**Keywords:** BERT, Explainable AI, Multi-objective optimization, Neurological medicines, Neuropeptides, NSGA-II, Machine learning, Protein design

## Abstract

The advent of the fourth industrial revolution, characterized by artificial intelligence (AI) as its central component, has resulted in the mechanization of numerous previously labor-intensive activities. The use of in silico tools has become prevalent in the design of biopharmaceuticals. Upon conducting a comprehensive analysis of the genomes of many organisms, it has been discovered that their tissues can generate specific peptides that confer protection against certain diseases. This study aims to identify a selected group of neuropeptides (NPs) possessing favorable characteristics that render them ideal for production as neurological biopharmaceuticals. Until now, the construction of NP classifiers has been the primary focus, neglecting to optimize these characteristics. Therefore, in this study, the task of creating ideal NPs has been formulated as a multi-objective optimization problem. The proposed framework, NPpred, comprises two distinct components: NSGA-NeuroPred and BERT-NeuroPred. The former employs the NSGA-II algorithm to explore and change a population of NPs, while the latter is an interpretable deep learning-based model. The utilization of explainable AI and motifs has led to the proposal of two novel operators, namely p-crossover and p-mutation. An online application has been deployed at https://neuropred.anvil.app for designing an ideal collection of synthesizable NPs from protein sequences.

## Introduction

The fourth industrial revolution, kick-started by artificial intelligence (AI) and the Internet of Things (IoT), has resulted in the rampant development of machine learning (ML) and deep learning (DL) to replace human expertise in performing numerous tasks. As a result, the applications of ML and DL have been growing by leaps and bounds. One such application is protein analysis^1^, and an emerging application regarding this is the development of an in silico tool to discriminate between therapeutic and non-therapeutic peptides (peptides are small chains of amino acid (AA) residues that form a protein)^2–9^. This could lead to the synthesis of new and improved drugs. However, just building a model to classify and discover such peptides in proteins is not sufficient for helping wet-lab researchers decide which peptides should be preferred for synthesis. Moreover, over-reliance on only the model’s predicted probability (MPP) value for making such a decision is too risky since this could waste time, effort, and resources. Therefore, there is a need for robust models that consider the vital properties of therapeutic peptides (that make them desirable for chemical synthesis) while giving results. These properties can be used to frame a multi-objective optimization (MOO) problem^10^, which can be solved using AI tools and techniques.

Finding an optimal solution to MOO problems with one or more conflicting objectives is not possible in polynomial time when the search space for solutions is too large to be explored exhaustively^11^. This difficulty led to the development of robust algorithms that can effectively explore the search space in a meaningful amount of time and provide us with an acceptable set of solutions with a reasonable trade-off amongst the given objectives (known as Pareto-optimal solutions). Nowadays, population-based meta-heuristic techniques are being employed for this purpose, among which the non-dominated sorting-based genetic algorithm (NSGA) is widely used. It outputs a Pareto-optimal set of non-dominated solutions based on a given set of objectives^12^. Deb et al. proposed NSGA-II^13^ as an improvement over NSGA after proving that it has reduced time complexity and is elitist as it saves the best solutions in every generation. Thus, it can be used to build an effective model based on a given set of objectives and properties to discover desirable therapeutic peptides in a vast search space.

Neuropeptides (NPs) are a class of therapeutic peptides actively being researched for their potential use in manufacturing biopharmaceuticals^14^ to cure neurological ailments. These are small biomolecules produced mainly by neurons that attach to G-protein-coupled receptors. This interaction is vital for various brain functions like modulating glucose metabolism, food intake, sleep cycles, and neuroprotection. It has been found that NPs such as ghrelin, neuropeptide Y, and pituitary adenylate cyclase-activating polypeptide play neuroprotective roles against various neurodegenerative disorders like Parkinson’s disease and Alzheimer’s disease^15^. However, there has been limited research on automating and accelerating the discovery of NPs. In^16^, authors built a meta-model named Neuropred-FRL using numerous baseline models. In another study^17^, authors proposed a stacked ensemble method in which the base-level consists of eight machine learning algorithms and the meta-level consists of a single classifier built using logistic regression (LR). NeuroPred-PLM^18^ also employs a multi-scale convolutional neural network for model construction. Lastly, in NeuroPIpred^19^, support vector machine (SVM) was used to classify neuropeptides. Although these models perform well on their respective datasets, they utilize hand-engineered features as their input. This is the first stage of dependence on human expertise that slows down the discovery of NPs. One can get rid of this bottleneck using deep learning techniques like recurrent neural networks (RNNs), long short-term memory networks (LSTMs)^20^, temporal convolutional networks (TCNs), transformers^21^, bidirectional encoder representations from transformers (BERT)^22^, etc., which automatically generate features from peptide sequences.

Moreover, the aforementioned studies have focused on building only simple classifiers for discriminating between NPs and non-NPs. Also, these classifiers are not explainable (they do not provide any information as to which features were crucial in classification). This leads us to the second stage of dependence on human experts. When a large set of NPs is predicted in a protein by a classifier, the results focus on the maximum MPP values only. Thus, there is a need for human expertise in deciding which NPs should be selected for chemical synthesis. It is a time-intensive process because not only do the wet-lab researchers assess a large pool of NPs, but also, sometimes, tweaking some AAs in peptides might result in an even better set of NPs. The selection decision is also taken after certain other properties of peptides, like the molecular weight, sequence similarity with the experimentally validated peptides, and presence of motifs (a motif is a short pattern of three or four AAs that recurs in many peptides of a particular class and its presence is purported to have some effect on their functional aspects), etc. In^23^, Liu et al. have considered evolutionary algorithms for finding a set of antimicrobial peptides (AMPs) with the classifier’s probability value and search space diversity as its objectives. However, these two objectives are insufficient to give a set of synthesizable peptides practically. In yet another study^24^, authors came up with an approach that uses a few properties, like molecular weight and sequence similarity score, among others, to measure a peptide’s bioactivity using NSGA-II. However, this work determines the desirability of a peptide on the basis of only a few handcrafted properties of peptides.

To alleviate the aforementioned difficulties and explore new possibilities, a novel model, NPpred, has been proposed in this work, which consists of two phases. In phase 1, an explainable deep learning tool called BERT-NeuroPred is developed^22^ to classify NPs using their sequence. Since deep learning algorithms generate and learn features independently, this model covers all the structural and functional aspects of a peptide. Also, the model has been made interpretable using Captum library^25^ to determine the AAs that contributed positively to a particular classification decision. A dataset of experimentally validated NPs and non-NPs collected from public databases has been used for training, tuning, and testing this model. The proposed model was compared with some state-of-the-art algorithms like NeuroPIpred^19^, NeuroPred-FRL^16^, and NeuroPred-PLM^18^ on the test set. After this, the MPP value given by BERT-NeuroPred for a peptide, along with its molecular weight and sequence similarity score given by Needleman Wunsch Algorithm (NWA) (with the experimentally validated NPs found in public databases), has been used to formulate a novel MOO problem. After this, phase 2 begins, in which these three objectives were used to determine non-dominated fronts and calculate the crowding distance based on a preference-based function. Moreover, to preserve motif-based information of peptides, the conventional mutation and crossover operations of NSGA-II have been modified to propose two novel operators termed p-mutation and p-crossover. Also, the explainable BERT-NeuroPred has been used while performing p-mutation. The optimization model built in phase 2 of this work is termed NSGA-NeuroPred, and together with BERT-NeuroPred, it forms the framework which has been named NPpred.

To sum up, the main contributions of this paper are summarised as follows.

- A novel framework called NPpred has been proposed to discover and optimize NPs while considering some desirable traits to enable faster synthesis of neurological biopharmaceuticals. It has two components, namely, BERT-NeuroPred and NSGA-NeuroPred. To our knowledge, this type of work has not been done before.
- A novel multi-objective optimization problem has been formulated to discover a Pareto-optimal set of NPs using NSGA-II, resulting in a framework termed NSGA-NeuroPred.
- A novel explainable deep learning model (BERT-NeuroPred) has been built for classifying neurological peptides. Thorough statistical tests using analysis of variance (ANOVA) and Tukey honestly significant difference (HSD) test have been performed to compare all the possible models that can be built using state-of-the-art machine/ deep learning techniques.
- A preference-based function has been proposed for calculating the crowding distance for assigning more priority to some objectives than others.
- Two novel peptide-specific operators, p-crossover and p-mutation, have been proposed by modifying the conventional crossover and mutation operators of NSGA-II.
- A free web app based on the proposed model has been deployed at https://neuropred.anvil.app to help wet-lab researchers discover a set of NPs with optimal MPP value, molecular weight, and NWA score.

The rest of the paper is organized as follows. Section “Background” contains a brief description of the tools and techniques used in this work. In section “Proposed approach”, the proposed model NPpred and its components have been thoroughly described and discussed. Section “Experiments, results, and discussions” presents the readers with details of the conducted experiments, and section “Experiments, results, and discussions” contains the discussion about the results. Lastly, we conclude our work and discuss its future scope in section “Conclusion”.

## Background

This section briefly describes the techniques and methods that were used for building the NPpred model.

### Non-dominated sorting genetic algorithm-II

NSGA is a metaheuristic based on genetic algorithms often employed to solve MOO problems^12^. It finds a Pareto-optimal front using a population of feasible solutions (where each solution is an *n*-dimensional vector comprising *n* variables) in a given search space. A general MOO problem can be expressed as per Eq. (16) (here, a multi-objective minimization problem has been considered), where *S* is a set of feasible solutions, $f_i$ is the *i*th objective such that 1 $<i \le k$, and $c_j$ is a set of constraints such that $1 < j \le h$.

1 $$\begin{aligned} {\textbf {Minimize : }} f (s) = {f_1 (s), f_2 (s), \dots , f_k (s)} \mid s \epsilon S \end{aligned}$$

2 $$\begin{aligned} {\textbf {Subject to : }} {c_1, c_2, \dots , c_h} \end{aligned}$$

A solution $s_1 \epsilon S$ dominates another solution $s_2 \epsilon S$ if and only if $s_1$ is either better than or equivalent to $s_2$ in the case of every objective and is better than $s_2$ for at least one objective (dominance of $s_1$ over $s_2$ can be represented as $s_2 \preceq s_1$ as given in Eq. (3)). Moreover, a solution $s_1$ is called Pareto optimal if and only if no other solution dominates $s_1$. A set of all Pareto optimal solutions is referred to as a Pareto optimal set *X* (which can be explained using Eqs. (3) and (5) for a minimization problem).

3 $$\begin{aligned} s_2 \preceq s_1 \implies \forall i : f_i(s_1) \le f_i(s_2) \end{aligned}$$

4 $$\begin{aligned} \wedge \exists i : f_i(s_1) < f_i(s_2) \mid 1 \le i \le k \end{aligned}$$

5 $$\begin{aligned} X = \{ s_1 \epsilon S \mid \forall s_2 \epsilon S : s_2 \preceq s_1\} \end{aligned}$$

NSGA-II^13^ is an improvement over NSGA because it is elitist (which means that, unlike NSGA, it saves the best solutions obtained in every iteration) and has O ($n^2$) time complexity (for simple NSGA, it is O ($n^3$)). The execution of NSGA-II involves certain specific steps. First, a parent population consisting of *N* solutions is initialized. Second, non-dominated sorting (NDS) is performed to assign ranks to all the solutions in the population (rank one is given to the best non-dominated solutions, rank two is assigned to the solutions dominated by solutions with rank 1, and so on). Third, a child population is generated by performing the crossover and mutation operations until the population has 2*N* solutions. Lastly, the combined parent and child populations are subjected to the selection operator by comparing their ranks and crowding distance (CD) (to estimate whether they are present in sparsely or densely populated regions in the search space). The crowding distance of an individual is computed as the sum of the distances between its immediate neighboring solutions along each objective. A solution with a high crowding distance means that it is located in a relatively sparsely populated region. Note that if two solutions have the same rank, the one with a high value of CD is preferred. Like this, a new population comprising *N* solutions is selected, and this process is repeated for a pre-specified number of generations (iterations).

### BERT

**Figure 1:**
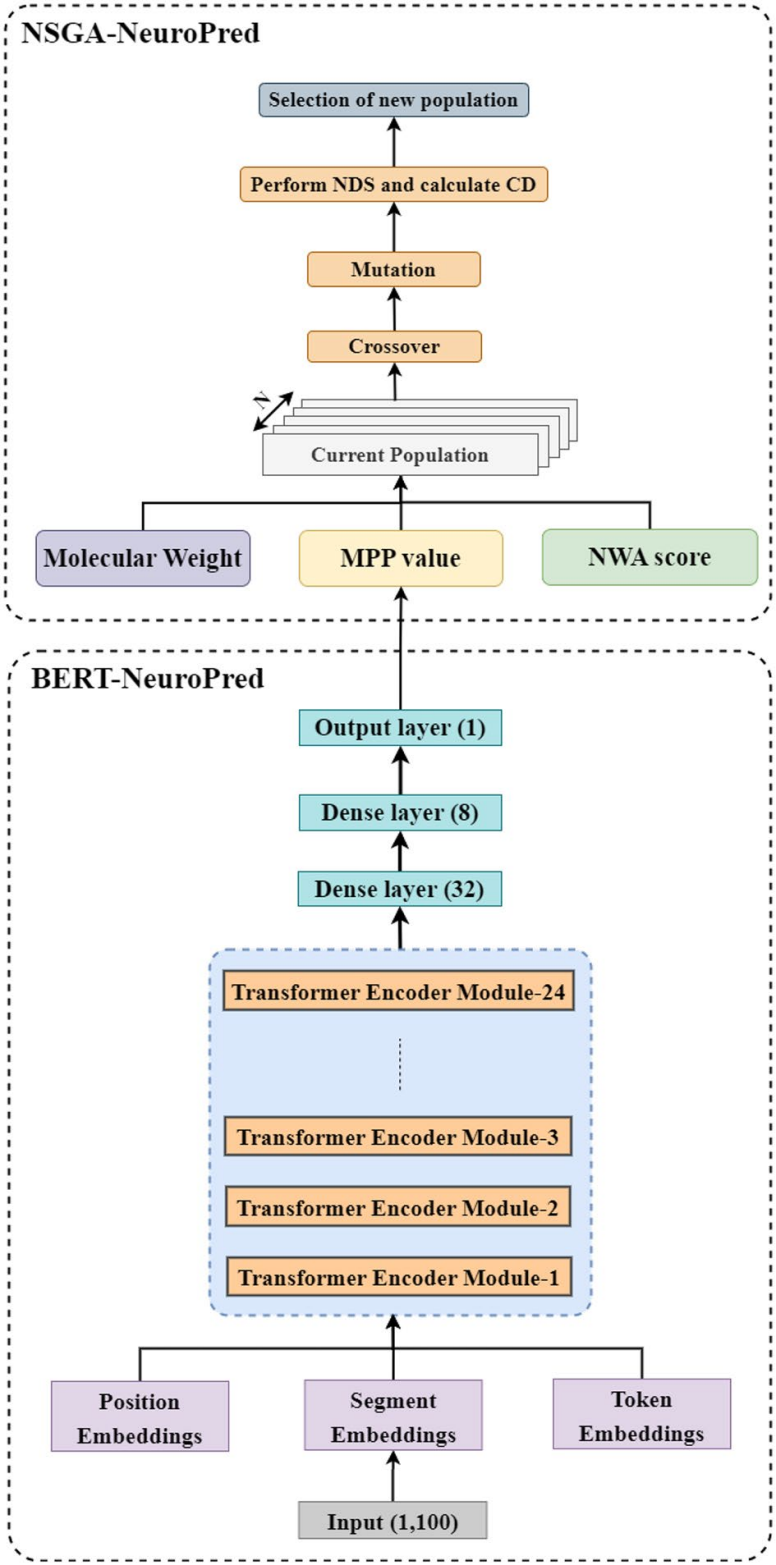
Model architecture of NPpred comprising BERT-NeuroPred and NSGA-NeuroPred.

BERT uses the transformers in a bidirectional manner using the masked language modeling technique. It is a great leap from the previously used language modeling techniques such as ELMo, GPT-2, etc., which either process a sequence in the forward or reverse direction or concatenate the result of both directions. BERT uses only the encoder module of the transformers, which learns bidirectional representations using both left and right contexts. In BERT, three types of embeddings are generated: token, position, and segment embeddings (as shown in Fig. 1). The token embeddings convert each word into a unique vector, the position embeddings contain information about the position of words in a given sequence, and the segment embeddings keep track of the sequence to which a particular token belongs. These embeddings are fed into an encoder module which consists of a multi-head attention mechanism and a point-wise fully connected layer. A multi-head attention module consists of multiple parallel self-attention mechanisms. Each self-attention mechanism uses query (*Q*), key (*K*), and value (*V*) vectors to calculate attention weights and scaled dot product attention (or *Att*, as given in Eq. (6), where $d_k$ is the dimension of key and query matrices) which helps in deciding the relative importance of words in a given sequence. The scaled-dot product attention of all self-attention mechanisms is concatenated together (as given in Eq. (7), where *MH-Att* stands for multi-head attention, $W_{i}^Q$, $W_{i}^K, W_{i}^V$, and $W^O$ are parameter matrices that are used to linearly project *Q*, *K*, *V*, and the final concatenated matrix, respectively and *h* is the number of self-attention heads) and fed into a linear layer to generate an enriched feature map for input sequences. The BERT-base and BERT-large pre-trained models use 12 and 24 encoder modules, respectively.

6 $$\begin{aligned} Att(Q, K, V)= \text {softmax} (\frac{Q.K}{\sqrt{d_k})}V \end{aligned}$$

7 $$\begin{aligned} MH-Att(Q, K, V)= \text { Concat }( Att (Q.W^{Q}_{1},K.W^K_{1},V.W^V_{1}), \end{aligned}$$

8 $$\begin{aligned} Att(Q.W^{Q}_{2},K.W^K_{2},V.W^V_{2}), \end{aligned}$$

9 $$\begin{aligned} \cdots , Att( Q.W^{Q}_{h},K.W^K_{h},V.W^V_{h}))W^O \end{aligned}$$

### Needleman Wunsch algorithm

This algorithm was developed to be applied in bioinformatics for aligning protein/peptide sequences and finding AA similarities in them^26^. It was among the first applications to use dynamic programming for comparing biological sequences. It is a global alignment and optimal matching technique for identifying similar regions within a pair of sequences that arise from or are a consequence of similar functional, structural, or evolutionary relationships. This algorithm explores every possible alignment between a given pair of peptides and returns the maximum similarity score, i.e., the one which is the result of optimum matching amongst them.

### Captum

Captum is a Python framework that provides tools for interpreting machine and deep learning models. Captum helps interpret and explain complex models, which may help understand the crucial factors behind their decisions. One of the techniques provided by Captum is integrated gradients, which establish a correlation between the output and the input features by integrating the gradients along a path from a baseline input to the actual input. In terms of sequence classification, it gives a positive or negative score to each component or token of the sequence as per their contribution to the final classification.

### Analysis of variance (ANOVA)

ANOVA is a statistical method used to compare the means of three or more groups to determine if there are statistically significant differences among them. It essentially tests the null hypothesis that all group means are equal against the alternative hypothesis that at least one group mean is different. ANOVA calculates and compares the F-statistic to a critical value from the F-distribution with appropriate degrees of freedom. If the calculated F-value is greater than the critical value, the null hypothesis is rejected, and it is concluded that there are significant differences between at least two group means. Then a post hoc test (e.g., Tukey’s HSD) can be conducted to identify which specific group means are different.

### Tukey’s honest significant difference (HSD) test

Tukey’s HSD test, often referred to simply as Tukey’s test, is a post hoc test commonly used after conducting an ANOVA to identify which specific group means differ from each other. It helps to determine which group pairs are significantly different from each other after finding a significant result in the ANOVA. The HSD value is calculated, and for each pair of group means, the difference is calculated and compared to this value. If the absolute difference between two group means is greater than the HSD value, then those two groups are considered significantly different from each other. Hence, the group that is better than the others can be found.

## Proposed approach

The proposed model NPpred involves building a deep learning classifier called BERT-NeuroPred for discriminating between NPs and non-NPs. Then, using the MPP value given by BERT-NeuroPred and two other objectives, a multi-objective minimization problem has been modeled for discovering a Pareto-optimal set of NPs using a population comprising NPs discovered by BERT-NeuroPred in neuro-proteins. This model is named NSGA-NeuroPred and is explained along with the BERT-NeuroPred model in this section.

### Phase 1: the BERT-NeuroPred model

In the first phase, a novel deep learning classifier was built and trained to accurately discriminate between NPs and non-NPs using the peptide sequences. To develop the best classifier, several deep and machine learning models were built and tested. Firstly, various deep and machine learning architectures like bi-LSTM, TCN, random forests (RF), SVM, gradient boosting (GB), and BERT were used for classifying NPs, and the models developed using them are given as follows.

#### Model_BERT

To build a sequence model using BERT, the pre-trained BERT-large-uncased model was employed. It consists of 24 sequential encoder modules with 16 attention heads each and a hidden size of 1024. The combined encoder module generates an embedding of size 1024, which is fed into a dense layer (D1) consisting of 32 neurons, followed by another dense layer (D2) consisting of 8 neurons. A sigmoid unit in the output layer gives the final classification. After fine-tuning the important hyperparameters such as batch size (16), learning rate (0.00001), optimizer (ADAM), and number of neurons in D1 (32) and D2 (8), the model was explained using the integrated gradients method of the Captum framework. The detailed representation of this model is shown in Fig. 1.

#### Model_biLSTM

For building this model, firstly, the skip-gram algorithm was used to generate feature vectors of size 256 for each amino acid. Then each peptide was represented using these features and input into a bi-LSTM layer with 200 units (neurons) and a drop-out rate of 10%. The feature map generated by this layer is fed into a global average pooling layer which converts it into a 1D tensor. This is followed by two sequential dense layers of sizes 64 and 16 and an output layer (with a single sigmoid unit).

#### Model_TCN

This model follows the same procedure to generate word embeddings like Model_biLSTM. Then, each peptide, represented using these word embeddings, is fed into a single TCN residual block with 100 units (neurons), consisting of dilations of sizes 1, 2, and 4, accompanied by layer normalization between the constituent 1D convolutional layers that form the TCN residual block. The feature maps generated by this layer are fed into a global average pooling layer, which converts it into a 1D vector. This is followed by two dense layers comprising 16 and 8 neurons and an output layer comprising a single sigmoid unit.

#### Stacked models

As discussed later in Section “Experiments, results, and discussions”, the Model_BERT outperformed both Model_TCN and Model_biLSTM. So, it was used as a baseline model on which some deep and machine learning layers were stacked to check the difference in performance. The features generated by it acted as input to a TCN block, SVM, RF, and GB, and the models generated were named Model_BERT + TCN, Model_BERT + RF, Model_BERT + SVM, and Model_BERT + GB, respectively.

### Phase-2: the NSGA-NeuroPred model

The NSGA-II has been used to find a Pareto-optimal set of NPs, and the population is initialized using the peptides discovered by BERT-NeuroPred in a given protein. The solutions have been encoded as peptides, which are strings of letters representing amino acid (AA) residues. The operations performed on them and the formulated problem have been discussed.

**Algorithm 1:**
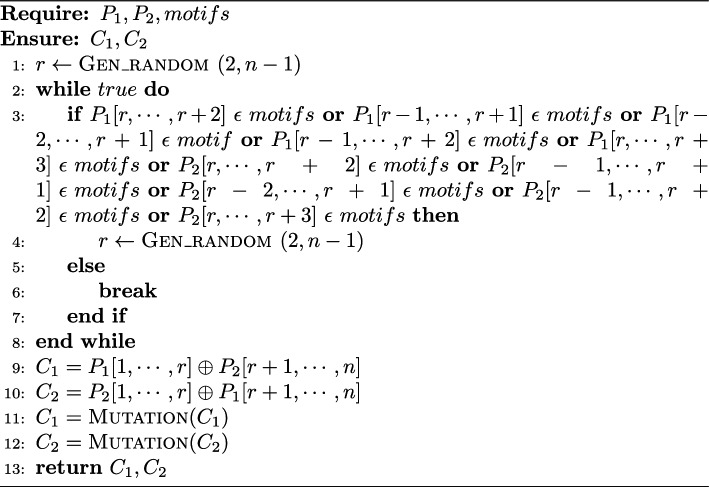
p-crossover

**Algorithm 2:**
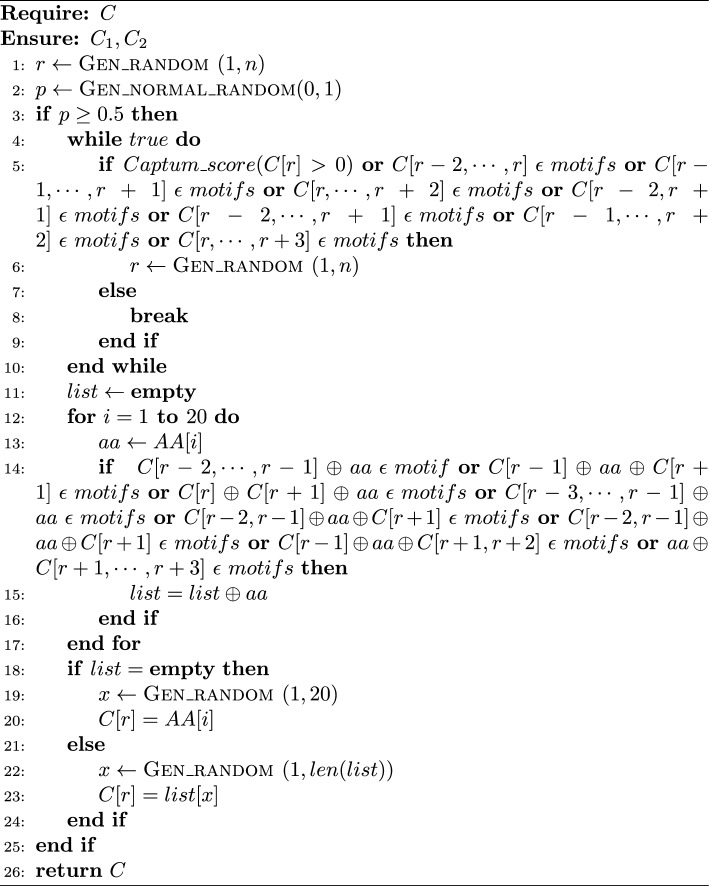
p-mutation

#### Problem formulation

This work optimizes three objectives: the MPP value given by BERT-NeuroPred (*MPP*), normalized NWA score with experimentally validated NPs ($nw_{{i}_{N}}$), and the normalized molecular weight $mw_{{i}_{N}}$. The objectives, in order of their importance, are explained as follows.

1. *Objective-1 (Probability score given by BERT-NeuroPred Model)* The BERT-NeuroPred model gives a predicted probability score for each peptide ($MPP_i$) in the population, which is a complex non-linear function. The higher the score, the higher the chance of a given peptide being neurological, as per the classifier. Thus, the value of this objective is to be maximized.
2. *Objective-2 (Needleman Wunsch Algorithm score)* NWA employs dynamic programming using the recursive relation given in Eq. (10) to provide a matching score for two sequences *Q* and *R* of lengths *m* and *n*, respectively. Here, $Sim(Q_{c},R_{d})$ is the similarity score for $Q\{1,\cdots ,c\}$ and $R\{1,\cdots ,d\}, F_{cd}$ is the NWA score for sequences $Q\{1,\cdots ,c\}$ and $R\{1,\cdots ,d\}$. The maximum score obtained after comparing a sequence *q* with all annotated peptides is considered as its NWA score ($nw\{Q,R\}$) (Eq. (11)).
  10 $$\begin{aligned} F_{cd}= & {} \max _{h<c,k<d} \{ F_{h,d-1}+Sim(Q_{c},R_{d}), F_{c-1,k}+Sim(Q_c,R_d)\} \end{aligned}$$
  11 $$\begin{aligned} nw\{Q,R\}= & {} F_{mn} \end{aligned}$$
  The NWA score ($nw_i$) for the $i^{th}$ sequence of the population ($s_i$) is calculated using Eq. (12). Here, the NWA score is calculated with every NP (*j*) present in the dataset *D*. The maximum matching score is considered as its NWA score. To standardize the scores for every sequence present in the population, the min-max standardization technique has been used, as given in Eq. (13). Here, the minimum and maximum possible matching scores are represented by $nw_{min}$ and $nw_{max}$, respectively.
  12 $$\begin{aligned} nw_{i}= {} \text {max}_{j \epsilon D} nw\{i,j\} \end{aligned}$$
  13 $$\begin{aligned} nw_{{i}_{N}}= {} (nw_i-nw_{min})/(nw_{max}-nw_{min}) \end{aligned}$$
3. *Objective-3 (Molecular weight)* Peptides with low molecular weight have a low manufacturing cost and high stability^27^. Thus, the lower a peptide’s molecular weight, the greater its therapeutic efficacy and feasibility of production. Whenever a peptide bond is formed, a water molecule is released. Thus, the molecular weight of a peptide is the sum of weights of the component AA residues minus the number of peptide bonds (which is one less than the number of AA residues) as given in Eq. (14). The molecular weight is standardized using Eq. 15, where $mw_{min}$ and $mw_{max}$ are the minimum and maximum weights associated with a peptide of length *l*.
  14 $$\begin{aligned} mw_{i}= & {} \sum ^{j=l}_{1}{mw_{AA_{j}}}-18.015*(l-1) \end{aligned}$$
  15 $$\begin{aligned} mw_{{i}_{N}}= & {} (mw_i-mw_{min})/(mw_{max}-mw_{min}) \end{aligned}$$

Neuropeptides with high MPP value, high NWA score, and low molecular weight are considered more desirable for production and therapeutic use. Hence, the NSGA-II has been used to find a Pareto-optimal front of non-dominated solutions (peptides) using these objectives. So, the minimization problem can be formulated as follows.

16 $$\begin{aligned} {\textbf {Minimize : }} f (i) = \{(1/MPP_i), (1/nw_{i_N}), mw_{i_N} \} \text {, }i \epsilon S \end{aligned}$$

#### Selection

The selection process in NSGA-II involves two steps: non-dominated sorting and crowding distance calculation. Non-dominated sorting divides the population into different fronts based on their non-dominated status. Individuals in the first front are non-dominated and are considered the best solutions (the Pareto-optimal front). Solutions in the first front dominate individuals in the second front, and so on. Crowding distance is used to ensure that the solutions selected for the next generation are diverse and spread across the front. It calculates the distance between individuals in each front and selects individuals that are in low-density regions to maintain diversity. After non-dominated sorting and crowding distance assignment, the individuals selected from each front are combined to form the population for the next iteration. This process ensures that NSGA-II converges to a set of solutions that are diverse and well-spread.

A preference-based function has been formulated for calculating the crowding distance as per Eq. (17). Here, rather than assigning the same priority to all objectives, some weights are assigned to each for calculating the CD for a given solution. More specifically, it is ensured that the MPP value is given more preference than the NWA score, which is given more priority than the molecular weight (as expressed in Eqs. (17) and (19)).

17 $$\begin{aligned} CD (i) = \alpha *(mpp_{(i+1)}-mpp_{(i-1)}) + \beta * (nw_{{(i+1}_{N})} \end{aligned}$$

18 $$\begin{aligned} -nw_{{(i-1}_{N})}) + \gamma * (mw_{{(i+1}_{N})}-mw_{{(i-1}_{N})}) \end{aligned}$$

19 $$\begin{aligned} \text {where }\alpha + \beta + \gamma = 1 \end{aligned}$$

20 $$\begin{aligned} \text {and } \alpha> \beta> \gamma >0 \end{aligned}$$

Here, $mpp_{(i-1)}$ and $mpp_{(i+1)}$ represent the MPP value of the solutions which come just before and just after the $i^{th}$ solution in a sorted list of MPP values. Similarly, $nw_{{(i-1}_{N})}$ and $nw_{{(i+1}_{N})}$ represent the NWA score of the solutions, which come just before and just after the $i^{th}$ solution in a sorted list of NWA values. Lastly, $mw_{{(i-1}_{N})}$ and $mw_{{(i+1}_{N})}$ represent the molecular weights of the solutions which come just before and just after the $i^{th}$ solution in a sorted list of molecular weights.

#### p-crossover

In general, a single point crossover involves randomly choosing a crossover point (CP) and swapping the substrings that lie to the right of the CP between the parents (as given in lines 9–10 of Algorithm 1). However, in this paper, the crossover operator has been modified, and a novel peptide-specific operator called p-crossover has been introduced, in which if the selected CP is in the middle of a three or four-AA motif (as per line 3 of Algorithm 1), the algorithm does not perform crossover at that point. Instead, it keeps looking for another CP until it finds the one that does not disrupt any motif in the parent peptides (line 4 of Algorithm 1).

#### p-mutation

The mutation operator has been modified into a novel operator called p-mutation. The working of p-mutation involves checking two conditions. Suppose an AA residue at a particular position *r* is selected for mutation/ replacement. As given in lines 5-6 of Algorithm 2, if either the AA to be replaced contributed positively to the classification by BERT-NeuroPred (as found using Captum), or it is part of a motif (of length 3 or 4), then it is not considered for mutation. The algorithm keeps selecting other positions (AA) until it finds the one whose mutation does not disrupt a motif, and also, it does not contribute positively to the classification. Next, as explained in lines 14-23 of Algorithm 2, the algorithm checks whether the AA residue present at *r* can be replaced with another AA residue in such a manner that it results in the formation of a three or four AA motif in the peptide. If there is no such possibility, the AA at *r* is replaced with a randomly chosen AA.

## Experiments, results, and discussions

This section describes the dataset and the details of the experiments conducted while building and testing the NPpred. The coding was done using Python; the models were trained on a compute node having 2.4 GHz Intel-Xeon Skylake 6148 CPU cores with 192 GB RAM and NVIDIA V100 GPU cores with 16 GB RAM. To build the model, Keras was used with Tensorflow^28^ as the backend. The pre-trained BERT model has been imported from the TensorFlow Hub. The models were evaluated using accuracy, F1-score, and area under the receiver operating characteristic curve (AUC).

### Dataset

The neuropeptides with 5 to 100 amino acid residues were collected from Neuropedia^29^, NeuroPep^30^, DiNer^31^, NeuroPIpred_DS1^19^, and Uniprot^32^. The non-NPs were obtained from the SwissProt database^33^ and SATPdb^34^. To eliminate similar sequences between the collected NPs and non-NPs, we performed CD-HIT-2D^35–37^ with a threshold of 0.9. Moreover, to prevent overestimation in performance due to homology bias, the CD-HIT^35–37^ has been applied to the NPs and non-NPs (separately) for eliminating the sequences with more than 90% sequence identity with one another. The dataset, which finally had 6807 NPs and 8647 non-NPs, was divided into training, validation, and test sets in the ratio 8:1:1 (80%, 10%, and 10%, respectively) for thorough experiments, analysis, and evaluation.

### Analysis of phase-1

In this paper, numerous experiments have been performed in which models were built using biLSTM, TCN, and BERT to find the one that performs the best on our test dataset. Moreover, various machine/ deep learning algorithms have also been applied to the features generated by the $\text {Model\_BERT}$, which led to the training and evaluation of many stacked models. These models were compared based on their mean performances on the given test set. A thorough analysis of variance (ANOVA) test was performed to interpret the statistical significance of the obtained results. This was followed by a post-hoc analysis using the Tukey’s honestly significant difference (HSD) test. The null hypothesis postulates that the mean performance of all the classifiers is more or less equivalent. If the p-value given by ANOVA is less than the selected alpha level (0.05), it means that the null hypothesis is false and the mean performance of at least one classifier has a statistically significant difference vis-a-vis other classifiers. To find the groups that differ from others in their mean performance, the Tukey HSD test was performed, which conducts a pair-wise comparison for all the classifiers. In this test, the mean performances of those pairs are considered to have a statistically significant difference for whom the adjusted p-value is lesser than the alpha level (0.05), and the 95% confidence interval (CI) (that lies between the Lower bound (LB) and Upper bound (UB) given by the test) does not contain zero. If the difference of means (meandiff) between group 1 and group 2 is less than zero, it implies that the mean of group 1 is higher than that of group 2.

#### Deep learning models

**Figure 2:**
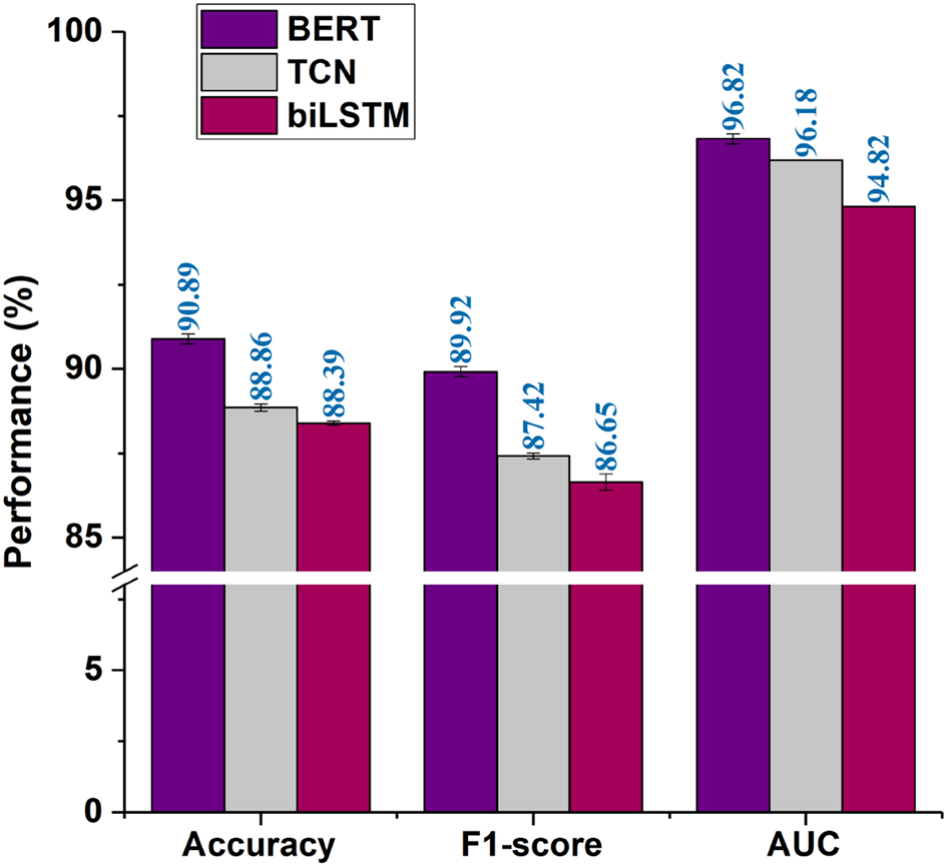
Performance of different deep learning models on the test set.

In this work, thorough experiments have been conducted by training and tuning three state-of-the-art deep learning algorithms, BERT, TCN, and biLSTM, on the training and validation sets. The resultant models were compared on the test set as illustrated in Fig. 2. It can be seen that $\text {Model\_BERT}$ performs the best in the case of all the metrics. Also, to test the statistical significance of these results, ANOVA and Tukey HSD tests were performed on the results furnished by these models as given in Tables 1, 2 and 3. It is clear that the p-value is less than 0.05 in the case of all three metrics. So, the difference in the mean performance of all three models is statistically significant. Further, on performing the Tukey HSD test, the value of p-adjusted is less than 0.05 in the case of all the pairs of models. Since the $\text {Model\_BERT}$ has the highest mean performance in the case of all the metrics, it concluded that it is the best classifier among all the three models.

**Figure 3:**
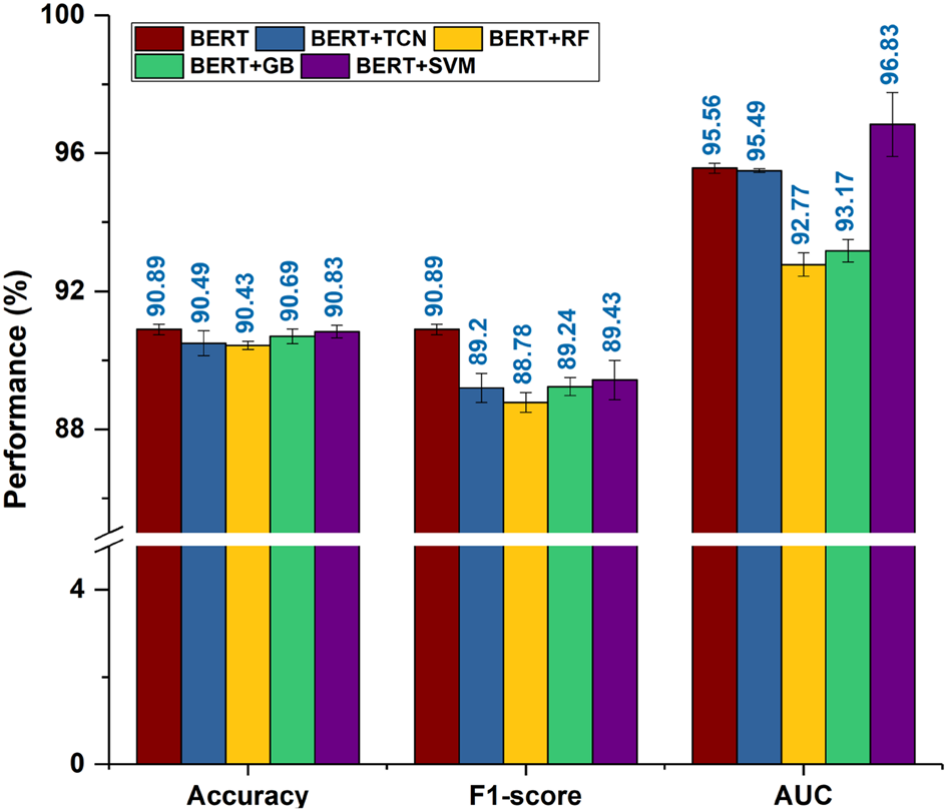
Performance of different stacked models on the test set.

**Table 1:** ANOVA on accuracy (%) of all the proposed models after training on ten different dataset splits.

(a) Input summary						
Group	Count	Sum	Average	Variance		
Model_BERT	10	908.89	90.89	0.15		
Model_TCN	10	883.98	88.39	0.08		
Model_biLSTM	10	888.59	88.86	0.11		

(b) ANOVA result						
Source of Variation	SS	df	MS	F-stat	P-value	F-crit
Between Groups	35.13	2	17.56	153.52	1.79E-15	3.35
Within Groups	3.09	27	0.11	–	–	–
Total	38.22	29	−	–	–	–

(c) Tukey HSD result						
Group 1	Group 2	Meandiff	p-adjusted	Lower-bound	Upper-bound	
Model_BERT	Model_TCN	− 2.03	0.00	− 2.41	− 1.65	
Model_BERT	Model_biLSTM	− 2.49	0.00	− 2.87	− 2.12	
Model_TCN	Model_biLSTM	− 0.46	0.01	− 0.84	− 0.09

**Table 2:** ANOVA on F1-score (%) of all the proposed models after training on ten different dataset splits.

(a) Input summary						
Group	Count	**Sum**	Average	Variance		
Model_BERT	10	899.18	89.92	0.15		
Model_TCN	10	874.16	87.42	0.09		
Model_biLSTM	10	866.50	86.65	0.24		

(b) ANOVA result						
Source of variation	SS	df	MS	F-stat	P-value	F-crit
Between Groups	58.42	2	29.21	178.56	2.71E-16	3.35
Within Groups	4.42	27	0.16	–	–	–
Total	62.84	29	–	–	–	–

(c) Tukey HSD result						
Group 1	Group 2	Meandiff	p-adjusted	Lower-bound	Upper-Bound	
Model_BERT	Model_TCN	− 2.50	0.00	− 2.95	− 2.05	
Model_BERT	Model_biLSTM	− 3.27	0.00	− 3.72	− 2.82	
Model_TCN	Model_biLSTM	− 0.77	0.00	− 1.21	− 0.32

**Table 3:** ANOVA on AUC (%) of all the proposed models after training on ten different dataset splits.

(a) Input summary						
Group	Count	Sum	Average	Variance		
Model_BERT	10	968.16	96.82	0.15		
Model_TCN	10	961.81	96.18	0.01		
Model_biLSTM	10	948.22	94.82	0.01		

(b) ANOVA result						
Source of variation	SS	df	MS	F-stat	P-value	F-crit
Between Groups	20.75	2	10.37	181.53	2.20E-16	3.35
Within Groups	1.54	27	0.06	–	–	–
Total	22.29	29	–	–	–	–

(c) Tukey HSD result						
Group 1	Group 2	Meandiff	p-adjusted	Lower-bound	Upper-Bound	
Model_BERT	Model_TCN	− 0.64	0.00	− 0.90	− 0.37	
Model_BERT	Model_biLSTM	− 1.99	0.00	− 2.26	− 1.73	
Model_TCN	Model_biLSTM	− 1.36	0.00	− 1.62	− 1.09

**Table 4:** ANOVA on accuracy (%) of all the proposed models after training on ten different dataset splits.

(a) Input summary						
Group	Count	Sum	Average	Variance		
Model_BERT	10	908.89	90.89	0.15		
Model_BERT + TCN	10	904.88	90.49	0.36		
Model_BERT + RF	10	904.34	90.43	0.12		
Model_BERT + GB	10	906.96	90.69	0.21		
Model_BERT + SVM	10	908.25	90.83	0.19		

(b) ANOVA result						
Source of variation	SS	df	MS	F-stat	P-value	F-crit
Between Groups	29.34	5	5.87	7.88	1.25E−05	2.39
Within Groups	40.22	54	0.74	–	–	–
Total	69.56	59	–	–	–	–

(c) Tukey HSD result						
Group 1	Group 2	Meandiff	p-adjusted	Lower-bound	Upper-Bound	
Model_BERT	Model_BERT + GB	− 0.19	0.87	− 0.76	0.37	
Model_BERT	Model_BERT + RF	− 0.45	0.17	− 1.02	0.11	
Model_BERT	Model_BERT + SVM	− 0.06	0.99	− 0.63	0.50	
Model_BERT	Model_BERT + TCN	− 0.40	0.28	− 0.97	0.17

**Table 5:** ANOVA on F1-score (%) of all the proposed models after training on ten different dataset splits.

(a) Input summary						
Group	Count	Sum	Average	Variance		
Model_BERT	10	899.18	89.92	0.15		
Model_BERT + TCN	10	892.01	89.20	0.42		
Model_BERT + RF	10	887.83	88.78	0.29		
Model_BERT + GB	10	892.38	89.24	0.26		
Model_BERT + SVM	10	893.41	89.34	0.57		

(b) ANOVA result						
Source of variation	SS	df	MS	F-stat	P-value	F-crit
Between Groups	6.64	4	1.66	4.89	0.002	2.58
Within Groups	15.25	45	0.34	–	–	–
Total	21.89	49	–	–	–	–

(c) Tukey HSD result						
Group 1	Group 2	Meandiff	p-adjusted	Lower-bound	c	
Model_BERT	Model_BERT + GB	− 0.68	0.08	− 1.42	0.06	
Model_BERT	Model_BERT + RF	− 1.138	0.00	− 1.88	− 0.39	
Model_BERT	Model_BERT + SVM	− 0.58	0.19	− 1.32	0.16	
Model_BERT	Model_BERT + TCN	− 0.72	0.06	− 1.46	0.02

**Table 6:** ANOVA on AUC (%) of all the proposed models after training on ten different dataset splits.

(a) Input summary						
Group	Count	Sum	Average	Variance		
Model_BERT	10	968.16	96.82	0.15		
Model_BERT + TCN	10	962.33	96.23	0.05		
Model_BERT + RF	10	927.66	92.77	0.34		
Model_BERT + GB	10	931.69	93.17	0.33		
Model_BERT + SVM	10	968.25	96.83	0.93		

(b) ANOVA result						
Source of variation	SS	df	MS	F-stat	P-value	F-crit
Between Groups	163.59	4	40.89	113.35	6.81E−23	2.58
Within Groups	16.24	45	0.36	–	–	–
Total	179.83	49	–	–	–	–

(c) Tukey HSD result						
Group 1	Group 2	Meandiff	p-adjusted	Lower-bound	Upper-bound	
Model_BERT	Model_BERT + GB	− 3.65	0.00	− 4.41	− 2.88	
Model_BERT	Model_BERT + RF	− 4.05	0.00	− 4.81	− 3.29	
Model_BERT	Model_BERT + SVM	0.01	1.00	− 0.75	0.77	
Model_BERT	Model_BERT + TCN	− 0.58	0.21	− 1.35	0.18	

#### Stacked models

For further analysis, different deep/machine learning layers were stacked on top of $\text {Model\_BERT}$ to check if any significant performance improvement could be obtained. For this purpose, algorithms like TCN, RF, GB, and SVM have been used, and the resultant models were named as $\text {Model\_BERT+TCN}$, $\text {Model\_BERT+RF}$, $\text {Model\_BERT+GB}$, and $\text {Model\_BERT+SVM}$. These models were compared on the test set, and their performances are depicted using Fig. 3. It can be seen that $Model\_BERT+SVM$ outperforms $\text {Model\_BERT}$ on AUC, but no other model seems to perform better than $\text {Model\_BERT}$. To test the statistical significance of the observed results, statistical analysis was performed, and the results given by these models are mentioned in Tables 4, 5 and 6 . It was observed that stacking different types of deep learning layers on the top of the BERT module led to only a slight variation in the baseline model’s performance (as shown in Fig. 3). As compared to $\text {Model\_BERT}$, the $\text {Model\_BERT+SVM}$ performs better in terms of AUC and worse in terms of F1-score and accuracy. Other models have either equivalent or worse performance than $\text {Model\_BERT}$ in the case of all the performance metrics. To decide whether stacking additional layers on top of the baseline model is required or not, it was checked whether there is any statistically significant difference in the mean performances of these models with respect to $\text {Model\_BERT}$. The results are shown in Tables 4, 5 and 6 and interpreted as follows.

1. *Accuracy:* The p-value is greater than 0.05 (Table 5(b)), implying that the difference in means is statistically significant for some models. On performing the Tukey’s test, it was found that all the stacked models have comparable means with $\text {Model\_BERT}$ since the adjusted p-value is greater than 0.05 in all the cases. Due to this, the 95% CI contains zero in all the cases.
2. *F1-score:* The p-value is greater than 0.05 (Table 4 (b)), implying that the difference in means is statistically significant for some models. On performing the Tukey’s test, it was found that all the models have comparable means with $\text {Model\_BERT}$ except for $\text {Model\_BERT+RF}$ where $\text {Model\_BERT}$ performs better than $\text {Model\_BERT+RF}$.
3. *AUC:* The p-value was lesser than 0.05 in this case also (Table 6(b)). Further, the Tukey’s test confirms that the difference of means of $\text {Model\_BERT}$ with $\text {Model\_BERT+TCN}$ and $\text {Model\_BERT+SVM}$ is statistically significant. When compared to $\text {Model\_BERT+TCN}$, $\text {Model\_BERT}$ performs better. On the other hand, it has lower performance than $\text {Model\_BERT+SVM}$. In the case of the remaining models, the $\text {Model\_BERT}$ has more or less equivalent performance.

Since the baseline model’s performance did not increase significantly after incorporating various machine/ deep learning layers, it was finally named BERT-NeuroPred and used for classifying the NPs. Following this, the results of the proposed model were explained using Captum in order to be used by the p-mutation operator. Furthermore, this model was compared with some state-of-the-art algorithms like NeuroPIpred^19^, NeuroPred-FRL^16^, and NeuroPred-PLM^18^ on the test set. The results (in Table 7) show that the overall performance of BERT-NeuroPred is better than all these models. The BERT-NeuroPred lags behind the NeuroPred-PLM in the case of recall but performs much better in the case of all other metrics. Also, the precision of NeuroPred-FRL is a bit higher but the values of all the other metrics are much lower than that of the proposed model.

**Table 7:** Comparison of BERT-NeuroPred with existing state-of-the-art (SOTA) models.

Model	Accuracy(%)	F1-score(%)	Precision(%)	Recall(%)	AUC(%)
**BERT-NeuroPred**	**91.14**	**90.08**	**90.64**	**89.34**	**97.12**
Neuropred-PLM	88.24	87.96	81.29	95.52	88.95
NeuroPred-FRL	87.27	84.83	91.07	79.39	93.84
NeuroPIpred	61.82	53.86	58.77	49.71	60.69

Significant values are in bold.

### Analysis of phase-2

**Figure 4:**
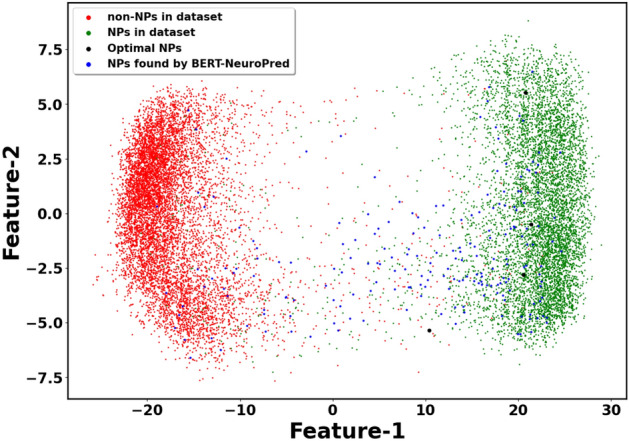
Isometric mapping of peptides found by NSGA-NeuroPred.

In the second phase, a web app was built and deployed at https://neuropred.anvil.app. Using this app, 205 neuropeptides were discovered (with MPP value $\ge$ 0.90 and 20 AA residues) in three well-known neuroproteins obtained from UniProt. These peptides were used to initialize the population on which NSGA-NeuroPred was executed for 50 generations. Using the isometric mapping technique, the distribution of peptides present in the dataset was visualized with the NPs found by BERT-NeuroPred (with MPP values $\ge$ 0.9) and the NPs present in the Pareto-optimal set found by NSGA-NeuroPred (Fig. 4). It can be observed that all three aforementioned groups of NPs (NPs found by BERT-NeuroPred, NSGA-NeuroPred, and the ones in the dataset) follow a similar distribution. It is noteworthy that the Pareto-optimal NPs, as given by NPpred, are well-dispersed and, at the same time, concentrated in the core region of the distribution of the experimentally validated NPs. This shows that NSGA-NeuroPred improves the results obtained by BERT-NeuroPred and also provides a wet lab researcher with a smaller and filtered set of NPs, which can be chemically synthesized and experimentally validated.

## Conclusion

In this work, an explainable deep learning and genetic algorithm-driven technique for accelerating the discovery and synthesis of neurological peptides (NPs) has been proposed. Apart from building a classifier, molecular weight and similarity with experimentally verified NPs were considered to model a MOO problem to explore the solution search space of NPs discovered by the classifier. In phase 1, an NP/ non-NP classifier called BERT-NeuroPred is built. The MPP value given by it was utilized with the NWA score and molecular weight for framing a minimization problem and for calculating the crowding distance using a preference-based function. This MOO module is named as NSGA-NeuroPred. Also, two novel operators called p-crossover and p-mutation were proposed. The combination of the BERT-NeuroPred and NSGA-NeuroPred model is named NPpred, and a free web app based on it has been deployed at https://neuropred.anvil.app. As a pilot study of this app, three well-known neuroproteins were entered, and NPs of length 20 were discovered. The NPs in the Preto-optimal front were shown to lie in the core region of the experimentally validated NPs’ and can be chemically synthesized and tested for their neurological activity.

There are certain limitations of the proposed model. Firstly, only a few characteristics were considered as objectives. Hence, in the future, various other secondary and tertiary characteristics of peptides, like isoelectric point, amphipathicity, etc., can be incorporated into the list of objectives. Also, a method to find the desirable optimizable characteristics can be designed to avoid a rigorous literature review for the same. Secondly, the model can be re-designed to find the NPs that can quickly form a peptide drug conjugate with drug carriers like blood-brain barrier penetrating peptides so that the resultant bio-pharmaceutical can easily reach its target in the brain. Lastly, other sequence modeling algorithms and model compression techniques can be explored to build better and faster classifiers since large language models such as BERT consume a lot of resources and hence the execution of the classifiers (including the proposed model) built using them is slow.

## Data Availability

The datasets analyzed during the current study will be made available upon reasonable request to the authors of this study. Vishakha Singh can be contacted to request the data used in this study.
